# Relationship and associated mechanisms between ambulatory blood pressure and clinic blood pressure with prevalent cardiovascular disease in diabetic hypertensive patients

**DOI:** 10.1097/MD.0000000000006756

**Published:** 2017-04-21

**Authors:** Zirui Hao, Guiping Li, Yue Sun, Yan Liu

**Affiliations:** The Third People's Hospital of Huizhou, Huizhou, Guangdong Province, China.

**Keywords:** aldosterone, ambulatory blood pressure, cardiovascular disease, clinic blood pressure, relationship

## Abstract

The present study was to compare the association between ambulatory blood pressure (ABP) and clinic BP (CBP) with prevalent cardiovascular diseases (CVD); and the underlying mechanism would also be investigated concurrently.

Diabetic hypertensive patients were enrolled and divided into 2 groups based on presence of CVD. Twenty-four hour-ABP monitoring was performed and between-group differences were evaluated and logistic regression analysis was conducted.

A total of 568 diabetic hypertensive patients were enrolled, and the mean age was 60.8 years, male accounted for 67.8%. Mean durations of diabetes mellitus and hypertension were 6.1 ± 2.7 and 5.4 ± 3.3 years, respectively, and 20.6% had prevalent CVD. Compared to patients without CVD, patients with CVD had significantly higher body mass index (BMI), plasma aldosterone concentration (PAC), and serum sodium level. No significant between-group differences in CBP were observed. However, 24 hour-SBP, daytime-SBP and nighttime-SBP were all significantly higher in patients with CVD compared to those without CVD. Pearson correlation analysis showed that BMI was positively correlated with PAC and serum sodium level. Logistic regression analyses showed that the association between clinic SBP and DBP with CVD were progressively attenuated to nonsignificant. In contrast, both ambulatory SBP and DBP were independently associated with CVD. However, after being further adjusted for PAC, no significant association was observed between ambulatory SBP and CVD.

In diabetic hypertensive patients, ABP is superior to CBP in relation to CVD. The association between ambulatory SBP and CVD may be dependent on aldosterone excess.

## 1 Introduction

Diabetes mellitus is a major public health problem around the world owing to its causal relationship with a variety of micro- and macro-vascular diseases.^[1,2]^ In the recent decades, several cross-sectional studies and longitudinal cohort studies have consistently showed that patients with diabetes mellitus have a higher incidence and prevalence of hypertension than those without diabetes mellitus.^[3]^ Furthermore, diabetic patients with hypertension are at increased risk of experiencing cardiovascular events than their diabetic counterparts without hypertension.^[3,4]^ Therefore, previous guidelines recommended that in diabetic patients with hypertension, blood pressure (BP) level should be reduced to lower than 130/80 mm Hg.^[5]^ Indeed, in the past few years, several high-quality meta-analyses have shown that systolic BP (SBP) level lower than 130 mm Hg was associated with lower cardiovascular events,^[6,7]^ despite the ACCORD-BP trial showed no statistical significant differences in the composite cardiovascular outcomes between the intensive (SBP <120 mm Hg) and the standard BP (SBP <140 mm Hg) groups.^[8]^

Ambulatory blood pressure monitoring (ABPM) has been recognized as a more accurate and comprehensive BP measurement modality compared to the clinic BP (CBP).^[9]^ ABPM could provide data on 24 hours, daytime, and nighttime BP as well as data on BP patterns.^[9]^ Previously, several studies have shown that in the general population, BP values obtained from ABPM were more closely associated with cardiovascular diseases (CVD) than that from CBP.^[10–12]^ Similarly, some observational studies also have shown that ambulatory blood pressure (ABP) was superior to CBP in relation to CVD in diabetic populations.^[13–15]^

Aldosterone excess was positively associated with body mass index (BMI) in hypertensive patients.^[16]^ Diabetic patients commonly have a higher prevalence of central obesity and higher BMI than the general populations.^[17]^ We thus hypothesized that aldosterone excess might be the potential mechanisms in relation to CVD in diabetic hypertensive patients. In our present study, we were going to evaluate the following aspects. On the one hand, we would compare whether ABP was superior to CBP in relation to prevalent CVD in our Chinese diabetic hypertensive patients. On the other hand, we would evaluate whether this association was dependent on aldosterone excess.

## 2 Methods

### 2.1 Studied populations

Studied populations were enrolled in the Third People's Hospital of Huizhou from January of 2015 to August of 2016. The present study was approved by the Clinical Research Ethical Committee of the Third People's Hospital of Huizhou and informed consent was obtained from each participant. Inclusion criteria were documented diabetes mellitus and hypertension; and exclusion criteria were those with type 1 diabetes mellitus, secondary hypertension, severe renal dysfunction with estimated glomerular filtration rate (eGFR) ≤60 mL/min/1.73 m^2^, currently using aldosterone antagonist, or history of atrial fibrillation.

### 2.2 Demographic and anthropometric data

Constructed questionnaire was used to collect participant's demographic data including age, gender, cigarette smoking status, duration of diabetes and hypertension, prevalent CVD, and present medication usage. Briefly, prevalent CVD included coronary heart diseases based on computer tomography with contrast or coronary angiography and ischemic stroke based on computer tomography and typical clinical symptoms, and transient ischemic attack was excluded. The height and weight were measured by investigators, which were used to calculate the BMI (weight in kilograms divided by height in square meters).

### 2.3 Biochemical parameters

Biochemical parameters including fasting plasma glucose (FPG), glycated hemoglobin (HbA1c), total cholesterol (TC), triglyceride (TG), alanine aminotransferase (ALT), serum creatinine (Cr), sodium and potassium levels, and plasma aldosterone concentration (PAC) were evaluated using fasting venous blood.

### 2.4 BP measurements

Briefly, CBP measurements were conducted in accordance to the JNC-7 guideline recommendation.^[5]^ Patients sit quietly for 5 minutes with their back supported before BP measurement. Nondominant arm was selected and was placed on the desk, which was parallel to the level of heart. Appropriate cuff size was used, which could circle at least 80% perimeter of the arm. Three times measurements were performed with 1 minute interval and the last 2 BP readings were recorded and averaged for the CBP value. ABPM was subsequently conducted using model 90207 (Space-Labs Medical Inc., Redmond, WA). Nondominant arm was selected and BP measures were set at every 20 minutes during daytime (6:00–22:00) and every 30 minutes during nighttime (22:00–6:00). Dipping BP pattern was defined as nighttime/daytime SBP ratio <0.9 while nondipping BP pattern was the ratio ≥0.9.^[9]^

### 2.5 Statistical analysis

Continuous variables were presented as mean ± standard deviation (SD) and compared by the independent student *t*-test, and categorical variables were presented as number and percentage of cases and compared by the chi-square test. Pearson correlation analysis was performed to evaluate the relationship between BMI with PAC and serum sodium level. Logistic regression analysis was used and pertinent variables were entered into the models in a tiered fashion, and the odds ratio of prevalent CVD was calculated for standardized increment of 1-SD of each BP component. All analyses were conducted in the SPSS 17.0 (Chicago, IL). All *P* values were 2 sided, and statistical significance was defined as *P* <.05.

## 3 Results

### 3.1 General characteristics

A total of 568 diabetic patients with hypertension were enrolled. As shown in Table 1, the mean age was 60.8 years, male participants accounted for 67.8% (n = 385) and 36.3% (n = 206) of participants were present cigarette smoking. The mean durations of diabetes and hypertension were 6.1 ± 2.7 and 5.4 ± 3.3 years, respectively. The mean BMI was 25.4 ± 4.7 kg/m^2^. A total of 117 participants (20.6%) had prevalent CVD, including 48 myocardial infarction, 57 ischemic stroke, and 12 peripheral artery disease.

**Table 1:**
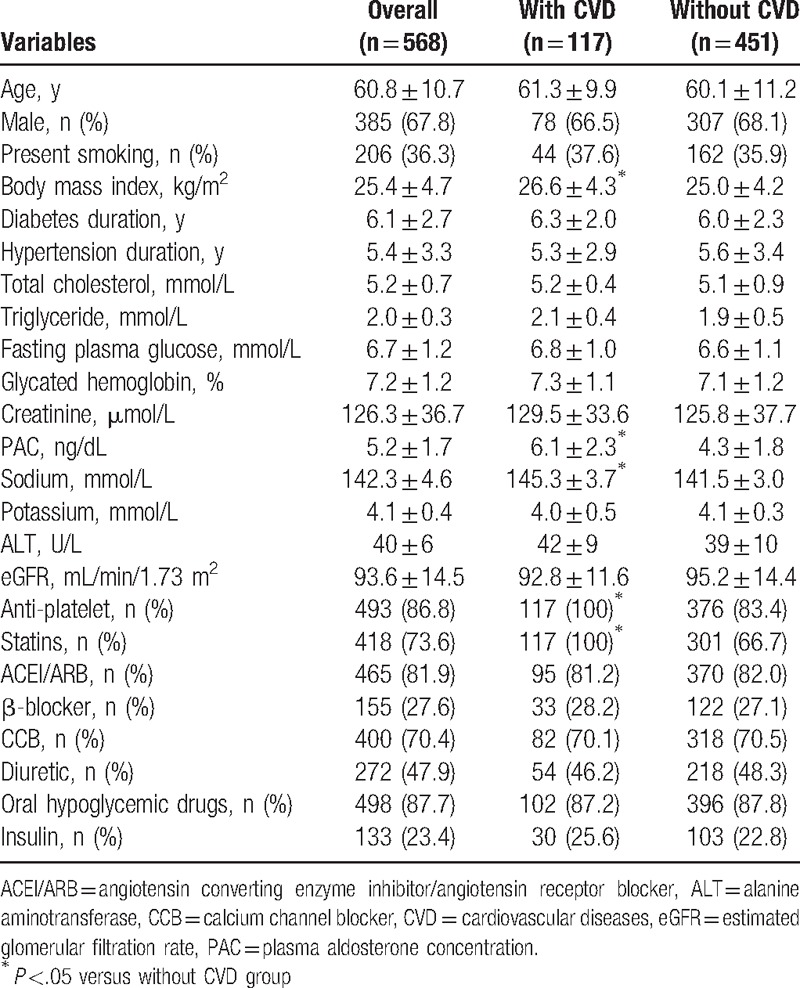
General characteristics and comparisons by categories of with- and without CVD.

### 3.2 Comparisons between subjects with- and without CVD

All participants were divided into with- and without CVD groups and between-group differences were evaluated. As shown in Table 1, compared to subjects without CVD, those with CVD had significantly higher BMI (26.6 ± 4.3 kg/m^2^ vs 25.0 ± 4.2 kg/m^2^), PAC (6.1 ± 2.3 ng/dL vs 4.3 ± 1.8 ng/dL), and serum sodium level (145.3 ± 3.7 mmol/L vs 141.5 ± 3.0 mmol/L). No significant differences in other variables such as antihypertensive medications and antidiabetic drugs usage were observed.

### 3.3 Comparisons of CBP and ABP by categories of with- and without CVD

**Figure 1:**
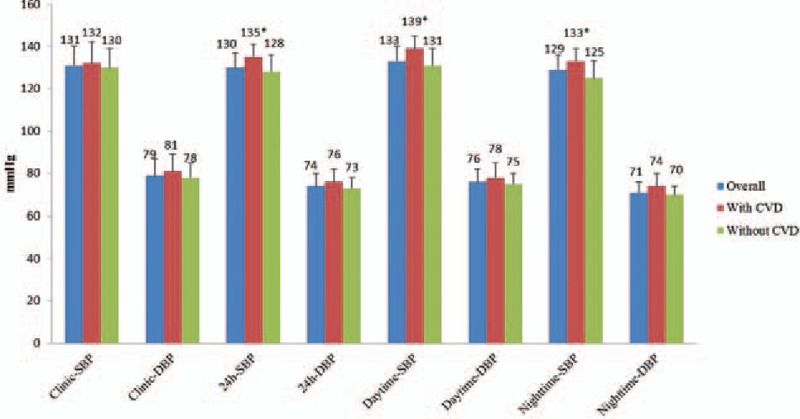
Comparison of BP components, ∗*P* <.05 versus without CVD group. BP = blood pressure, CVD = cardiovascular diseases.

As shown in Fig. 1, no significant between-group differences in clinic SBP and DBP were observed. However, with respect to ABP, 24 hour-SBP, daytime-SBP, and nighttime-SBP were all significantly higher in subjects with CVD than those without CVD (*P* <.05 for all comparisons). While no between-group differences in ambulatory DBP were observed. The nighttime/daytime SBP ratio was also significantly higher in subjects with CVD (0.95 ± 0.06 vs 0.91 ± 0.04, *P* <.05). The percentages of nondipping BP pattern in subjects with CVD versus without CVD were 73.5% (n = 86) and 63.6% (n = 287), respectively.

### 3.4 Pearson correlation analysis

**Figure 2:**
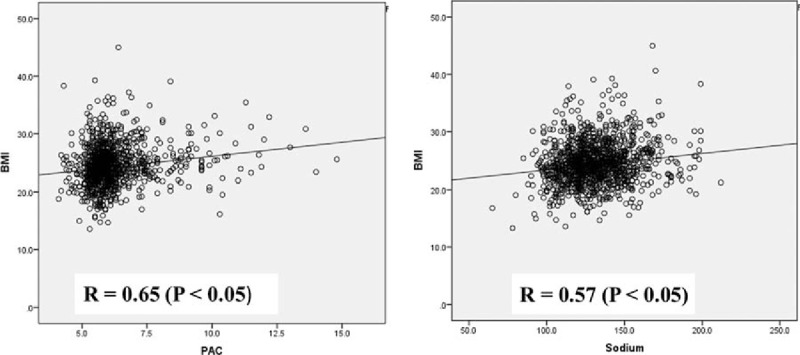
Relationship between BMI with PAC and serum sodium level. BMI = body mass index, PAC = plasma aldosterone concentration.

Pearson correlation analysis showed that BMI were positively correlated with PAC and serum sodium level, with a correlation coefficient of 0.65 and 0.57 (*P* <.05), respectively (Fig. 2).

### 3.5 Logistic regression analyses

Logistic regression analyses were performed to evaluate the association of CBP and ABP with prevalent CVD, respectively. As shown in Table 2, both clinic-SBP and clinic-DBP were not significantly associated with prevalent CVD after adjusted for age, gender, cigarette smoking, TC, HbA1c, and BMI. In 24 hour-SBP, daytime-SBP, and nighttime-SBP, after additionally adjusted for PAC, no significant association with prevalent CVD was observed. In 24 hour-DBP, daytime-DBP, and nighttime-DBP, after additionally adjusted for clinic-DBP, no significant association with prevalent CVD was observed.

**Table 2:**
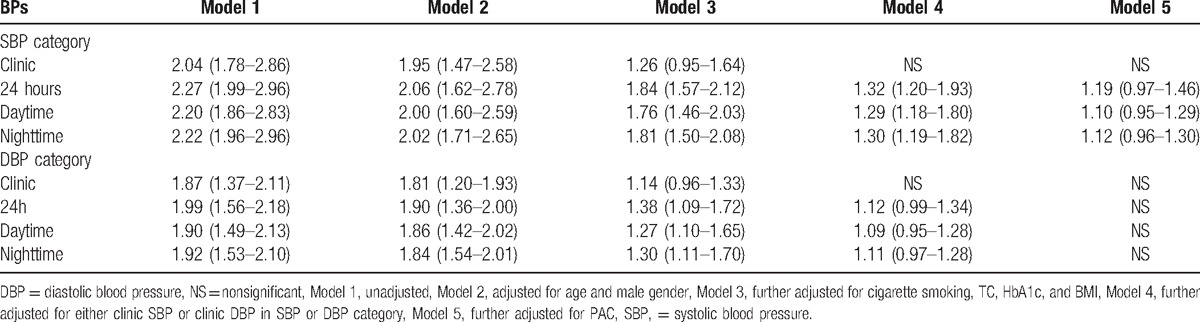
Logistic regression analysis (odds ratio and 95% confidence interval).

## 4 Discussion

Similar to prior studies,^[13,14,18]^ our present study also shows that in diabetic hypertensive patients, ABP is superior to CBP in relation to prevalent CVD. The novel finding of our present study is that aldosterone excess may be the underlying mechanism contributing to the association between ambulatory SBP and prevalent CVD in diabetic hypertensive populations. If confirmed that aldosterone excess is associated with increased incidence of cardiovascular events in diabetic hypertensive patients, using spironolactone may be beneficial for improving cardiovascular outcomes in these population groups in the future.

Diabetic patients with hypertension are at extremely high risk of experiencing cardiovascular events. Most of previous studies were focusing on investigating the optimal BP target. However, the results were inconclusive.^[8,19]^ One of the reasons might be the visit-to-visit variability of CBP. In addition, white-coat hypertension and masked hypertension might also influence the association between CBP and cardiovascular outcomes.^[20]^ Indeed, in recent decades, a substantial number of studies have shown the superiorities of ABP over CBP in relation to CVD. Similarly, in our present study, we also observed that subjects with CVD had significantly higher ambulatory SBP than those without CVD, although there was no significant difference in DBP between subjects with- and without CVD. Why there were no significant differences in ambulatory DBP was unclear. Knowingly, aging causes SBP elevation and DBP reduction,^[21]^ and the mean age of participants in our present study was 60.8 years, which represented an aging population. Therefore, we postulated that aging might be ascribed to this discrepant finding. Indeed, in Table 2, after adjusted for age and gender, the odds ratio was reduced more significantly in SBP category than that in DBP category, suggesting that age might play a substantial interactive role with SBP than with DBP. Future prospective study is warranted to investigate whether the differential effects of ambulatory SBP and DBP on incidence of CVD are truly dependent on age.

Interestingly and importantly, our present study showed that compared to those without prevalent CVD, subjects with CVD had significantly higher PAC. To our knowledge, this might be the first study to investigate the roles of aldosterone excess on diabetic hypertensive subjects. In a recent study, Dudenbostel et al^[16]^ revealed that PAC was positively correlated with BMI in subjects with resistant hypertension, suggesting that adipose-derived aldosterone might play an important role on BP elevation. Similarly, our present study also revealed a positive correlation between BMI and PAC, with a correlation coefficient of 0.65. Moreover, we also showed that after additionally adjusted for aldosterone, the association between ambulatory SBP and prevalent CVD was not significant, which might be because of aldosterone-induced fluid retention. Pathophysiologically,^[22]^ through increasing sodium and water retention, aldosterone excess elevates BP which in turn causes endothelial dysfunction and vascular damage. Indeed, in our present study, we also observed that compared to subjects without CVD, those with CVD had significantly higher serum sodium level.

There were several limitations of our present study. First of all, a cross-sectional design could not allow us to draw causal relationship. Second, this was a single-center study and whether our present findings could be extrapolated to other populations group was unknown. Third, as all participants were undergoing antihypertensive treatments during CBP and ABP measurements, the odd ratios of ABP and CBP for prevalent CVD might be underestimated. Fourth, the mean age of our participants was around 60 years and whether these findings could be extrapolated to the younger diabetic hypertensive populations was unclear.

In conclusion, our present study reveals that in diabetic hypertensive patients, ABP is superior to CBP in relation to prevalent CVD. Furthermore, aldosterone excess may be the potential mechanism contributing to the association between ABP and prevalent CVD. Future studies are needed to evaluate whether increased plasma aldosterone concentration could be used to predict CVD risk and whether aldosterone antagonist is beneficial for improving cardiovascular outcomes in these populations.

## Acknowledgments

Authors are indebted to Dr. David J Brown for his critical review of our paper.
